# Comparing active treatments at phase II: using multiple criteria to find the optimal treatment to take forward to phase III

**DOI:** 10.1186/1745-6215-14-S1-O117

**Published:** 2013-11-29

**Authors:** Michael Sweeting, Martin Law

**Affiliations:** 1 MRC Biostatistics Unit, Cambridge, UK

## 

In a Phase II RCT with multiple active treatments, we may often wish to take forward a treatment to Phase III based on multiple criteria and outcomes. In addition to efficacy, factors such as cost and the adverse event rate may play a key role in the decision making process. Using a Bayesian analysis, all relevant criteria can be considered, and a utility function employed to obtain the optimal treatment.

An example is presented, detailing the results of an RCT comparing Moxifloxacin, Doxycycline and Azithromycin against placebo in the treatment of COPD, using the criteria efficacy (reduction in airway bacterial counts), adverse event profile, treatment adherence, resistance to study drug and cost.

The relevant criteria of importance were chosen by the trial investigators. For each chosen criterion, a Bayesian analysis was performed and the effect estimates ranked, with uncertainty in the effect sizes automatically propagated to uncertainty in the ranks.

Each criterion was assigned a weight a priori, and these weights determined the relative importance of each criterion. The weights were chosen based on an elicitation exercise conducted with the clinicians, whereby possible outcomes were presented under various weighting scenarios in an iterative exercise until a consensus was achieved.

Finally, a utility function combined the elicited weights with the criteria ranks to provide an overall ranking for each treatment. The treatment recommended for Phase III was taken to be the one with the highest mean utility.

